# Mapping the current flow in sacral nerve stimulation using computational modelling

**DOI:** 10.1049/htl.2018.5030

**Published:** 2019-01-07

**Authors:** Nada Yousif, Carolynne J. Vaizey, Yasuko Maeda

**Affiliations:** 1 School of Engineering and Technology, University of Hertfordshire, Hatfield, AL10 9AB, UK; 2 Sir Alan Parks Physiology Unit, St Mark's Hospital, London, HA1 3UJ, UK; 3 Department of Surgery and Cancer, Imperial College London, London, SW7 2AZ, UK

**Keywords:** biomedical electrodes, prosthetics, patient treatment, neurophysiology, neuromuscular stimulation, bioelectric potentials, physiological models, biological tissues, finite element analysis, biomechanics, bioelectric phenomena, current flow, sacral nerve stimulation, computational modelling, established treatment, quadripolar electrode, sacral foramen, electrical stimulus, sacral nerve root, induced spread, electric current, SNS electrode, adjacent tissues, finite element model, biophysical models, nerve fibres, electrode model choice, contact configuration, electrode contacts, neural fibre stimulation, monopolar stimulation, bipolar stimulation, similar effect, therapeutic stimulation effects, adverse stimulation effects, stimulation parameters

## Abstract

Sacral nerve stimulation (SNS) is an established treatment for faecal incontinence involving the implantation of a quadripolar electrode into a sacral foramen, through which an electrical stimulus is applied. Little is known about the induced spread of electric current around the SNS electrode and its effect on adjacent tissues, which limits optimisation of this treatment. The authors constructed a 3-dimensional imaging based finite element model in order to calculate and visualise the stimulation induced current and coupled this to biophysical models of nerve fibres. They investigated the impact of tissue inhomogeneity, electrode model choice and contact configuration and found a number of effects. (i) The presence of anatomical detail changes the estimate of stimulation effects in size and shape. (ii) The difference between the two models of electrodes is minimal for electrode contacts of the same length. (iii) Surprisingly, in this arrangement of electrode and neural fibre, monopolar and bipolar stimulation induce a similar effect. (iv) Interestingly when the active contact is larger, the volume of tissue activated reduces. This work establishes a protocol to better understand both therapeutic and adverse stimulation effects and in the future will enable patient-specific adjustments of stimulation parameters.

## 1 Introduction

Faecal incontinence (involuntary leakage of stool and/or gas) is a socially embarrassing and stigmatising condition that affects 1 in 10 people [1]. This condition significantly impacts on the patients’ quality of life. There are various options available to treat faecal incontinence, which range from non-invasive conservative therapies to surgical options. For some patients, one of the established and least invasive surgical treatments is sacral nerve stimulation (SNS). SNS involves implanting electrodes into the patient's sacral foramen, through which an electric current is applied to the adjacent nerves [2]. The success rate in the short term is about 70–80% [3] and in the long-term, about half of patients treated by SNS derive some benefit from this treatment [4–6].

Obtaining such an improvement in symptoms depends on a number of factors, such as the precise placement of the electrode in the sacral nerve root region, choosing the best parameters for the stimulation and no movement of the hardware post implantation. These factors are, however, very hard to control post operatively and may be time consuming and laborious [7] and may not offer the optimal therapeutic settings. Furthermore, some patients develop side effects, such as pain, and some lose the beneficial effect over time [8, 9].

To address these limitations, we took a computational modelling approach to understand the link between parameter settings and the resulting electric field induced by SNS in the tissue surrounding the electrode. Such an approach has been used extensively in deep brain stimulation to visualise the impact of neuromodulation [10–12]. Two previous reports from the same group also used a similar computational modelling approach to studying SNS [13, 14], but in those works, the authors focused on transcutaneous electrodes alone and compared transcutaneous electrodes to implanted electrodes. In this study, we looked specifically at implanted electrodes for clinical SNS and at the comparison of two of the common models of electrodes, as well as contact configuration. By allowing the clinician to visualise the impact of each parameter setting, this approach has the potential to tailor therapy for each individual patient, and maximise the therapeutic effect whilst minimising adverse events such as pain and discomfort.

## 2 Methodology

### 2.1 Overview of the computational modelling approach

In order to construct our anatomical model of SNS, we took a three-step approach using a combination of the AC/DC (electrostatics) interface in COMSOL Multiphysics (3.5A) and Matlab (2009a). The first step involved segmenting MRI images in Matlab into two-dimensional representations of the sacrum and the colon on sequential slices, these, in turn, were converted into a three-dimensional geometrical model which was transferred into COMSOL Multiphysics. The second step was to implant an electrode and implanted pulse generator (IPG) in silica in the COMSOL FEM model and simulate SNS. Finally, the results of the electric potential distribution were exported from COMSOL and applied to axon models in the NEURON biophysical modelling package. Each of these steps is described in detail below.

### 2.2 Anatomical FEM model

**Fig. 1:**
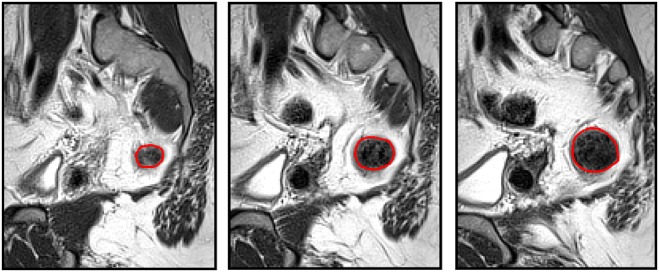
Segmentation process adopted to create the three-dimensional model. Three sequential sagittal slices of the dataset are shown, with the boundary of the segmented anatomical region, the colon, shown in red. These curves were then combined to form a three-dimensional model and the process repeated for the sacrum and the outer boundary of the body

First, a clinical pelvic MRI scan (T2 blade) of a female patient being investigated for rectal cancer, was used to provide the anonymised images for the SNS anatomical model. The image was chosen by a radiographer and the investigators had no access to the identity or details of the patient. We used 30 of these MRI slices (3.9 mm slice thickness, 1.15 mm × 1.15 mm voxel size) in the sagittal plane to form the three-dimensional model of the sacrum (seven slices were used as the sacrum appeared in only seven images of the MR dataset), the rectum (15 slices) and the pelvis (all 30 slices), which served as the outer boundary for our model. The anatomical regions were segmented from the atlas images using the function ‘roipoly’ in Matlab, by hand drawing the region to be segmented on each image. Fig. 1 shows a series of three such sagittal MRI images used in this process to construct the rectum and the region segmented on each image. In order to model the sacral foramen, the sacrum was not segmented as a single structure, but as multiple structures either side of and around the foramina in the bone.

**Fig. 2:**
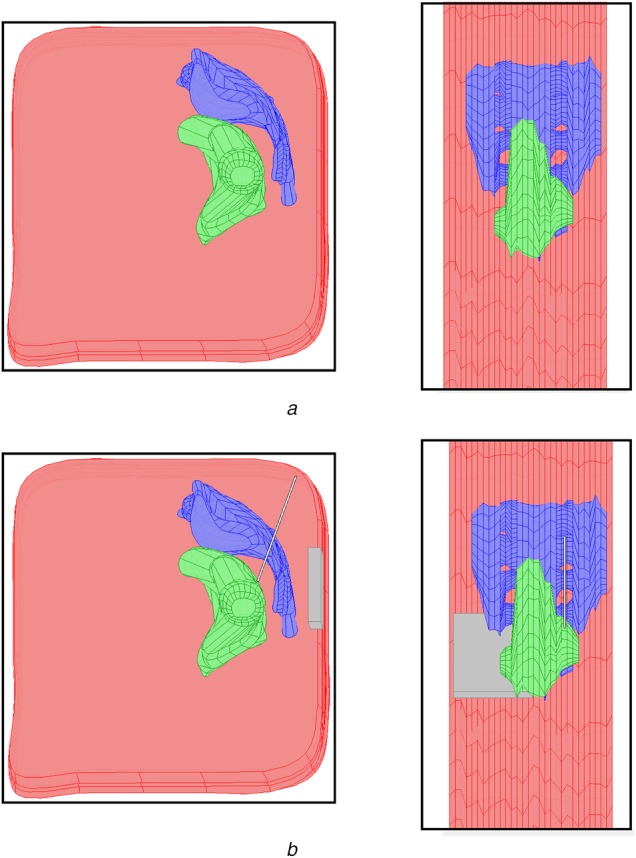
Four view of the three-dimensional finite element model created in COMSOL Multiphysics are shown *a* Top row gives a sagittal (left) and axial (right) view of the model with the three segmented anatomical regions: Pelvis (pink), rectum (green) and sacrum (blue) *b* Lower row shows the same views with the implanted electrode and IPG added to the model (grey)

For each anatomical structure, in turn, these two-dimensional regions of interest were converted into a three-dimensional solid using the COMSOL ‘loft’ command which smoothly fits a surface to a stack of two-dimensional cross sections. These three-dimensional solids were then imported into COMSOL, positioned correctly based on the MRI images and scaled based on the MRI resolution. The resulting three-dimensional model is shown in Fig. 2, where the separate parts of the sacrum have been fused into a single structure.

**Fig. 3:**
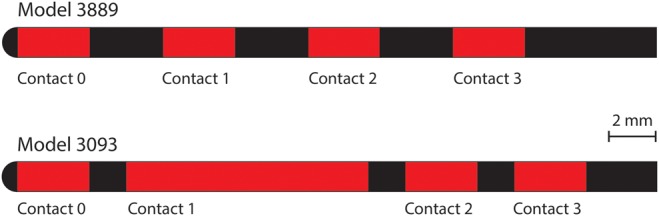
Schematic representation of the two models of quadripolar electrode represented in this study. Model 3889 (top), diameter of 1.27 and 3 mm long contacts separated by 3 mm. Model 3093 (bottom), diameter of 1.27 mm, three 3 mm long contacts and one 10.2 mm long contact, all separated by 1.5 mm

We then modelled and virtually implanted geometrically accurate representations of the two models of SNS electrode (Fig. 3). Note that we use the convention of referring to the contact nearest the tip as contact 0, followed by contacts 1–3. We simulated Medtronic model 3093, which has a diameter of 1.27 mm, and four contacts, three of which are 3 mm long and one of which (contact 1) is 10.2 mm long, all separated by 1.5 mm. We also simulated Medtronic model 3889, which has a diameter of 1.27 mm, and four contacts, which are 3 mm long, all separated by 3 mm. We also placed a geometrically representative IPG into the buttocks area via a cuboid measuring 55 mm by 60 mm by 10 mm. The IPG was placed at a location in the subcutaneous tissue under the skin, at a level representative of IPG locations in SNS patients, as measured from post-operative X-ray images.

The three different models were all meshed into tetrahedral elements using the default Delaunay meshing algorithm in COMSOL. The three models had the following mesh statistics: the homogeneous model had 93,613 elements; the anatomic 3093 electrode model had 484,743 elements and the anatomic 3889 model had 485,064 elements.

### 2.3 Theoretical analysis of SNS settings

We used a quasi-static FEM model (purely resistive) to simulate constant voltage SNS and to obtain the electrical potential distribution induced by each setting. The potential distribution induced by stimulation was calculated by solving the Laplace equation:

(1) $$\nabla .\sigma \nabla V = 0\eqno\lpar 1\rpar $$

where *V* is the electric potential, *σ* is the electrical conductivity, $\nabla $ is the gradient operator and $\nabla .$ is the divergence operator. The conductivity values were defined based on previous studies: bone 0.02 S/m, colon 0.01 and fat (used for the pelvic region) 0.01 S/m (measured at 14 Hz from http://niremf.ifac.cnr.it/tissprop/). The electrode and IPG were set as insulating boundaries, using Neumann boundary conditions so that current could not flow within these structures unless they were active during stimulation. For monopolar stimulation, one contact on the electrode was set to the cathodic stimulating voltage of 1 V and the IPG as the anodic return electrode using Dirichlet boundary conditions. For bipolar stimulation, one of the electrode contacts was the cathode and a second was the anodic return, with the IPG set as an insulated boundary. In all simulations, the outer boundary of the pelvis was also set to be insulated via Neumann boundary conditions.

### 2.4 Biophysical axon models

To quantify the effect of stimulation on nerves in the surrounding tissue, we used a previously described biophysical model of unconnected myelinated axons [15]. It is important to note that this approach is a state of the art method to quantitatively compare stimulation parameters. However, given the paucity of electrophysiological information about the sacral nerves, the model used here is an adapted existing model of myelinated axons. This model has been previously used to quantify the effects of the brain [16] and peripheral nerve stimulation [17] and is briefly described here. Double-cable models represent both the myelin sheath and the axolemma, with explicit representation of the nodes of Ranvier, paranodal and internodal segments. Implementing the models in NEURON v6.2, we used the 5.7 µm diameter axons, which contain a fast sodium conductance, a persistent sodium conductance, and a slow potassium conductance at the nodes. We extended the length of the axons to 100 mm to ensure we covered the extent of the targeted nerves.

**Fig. 4:**
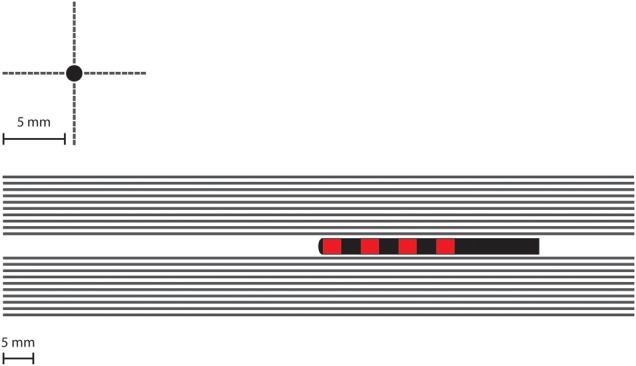
Location of the 40 modelled axons in relation to the electrodes and the contacts in two views. The axons are distributed to be parallel to the electrode direction to mimic the direction of nerve fibres in the sacrum. To quantify the impact of potential inaccuracy of the electrode relative to the nerve, ten fibres are placed superior to the electrode, ten inferior, ten lateral and ten medial

We modelled 40, 100 mm long axons in four rows of 10 axons, separated by 0.5 mm and with their long axis running parallel to the electrode to mimic the ideal trajectory of the electrode in SNS (Fig. 4). This arrangement was to mimic bundles of axons arranged in nerve fibres, however, does not represent the true separation of individual axons in sacral root bundles, as this would be well below the resolution of the FEM model and therefore would not yield more accurate results. The electrode is ideally placed running parallel to the nerve, however, the precise placement of the electrode relative to the nerve may be inferior, superior medial or lateral. Therefore to quantify the impact of each potential placement on the VTA, we modelled four locations of the nerve, superior, inferior, lateral and medial to the electrode. We used the potential distribution from the FEM model convolved with a time dependent square wave (14 Hz frequency, 1 V amplitude [18]) for 1000 ms as the extracellular stimulus delivered by SNS. The axons which fire in response to each stimulus pulse are taken as activated points in the surrounding neural tissue. Hence we delineated the so called volume of tissue activated (VTA).

## 3 Results

### 3.1 Homogenous model versus the anatomical model

**Fig. 5:**
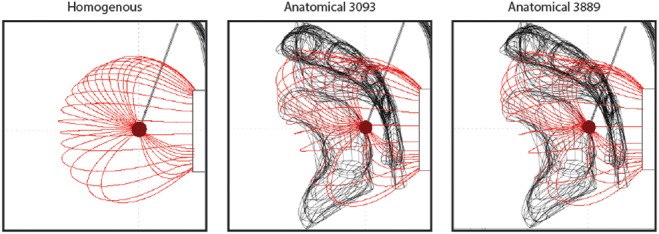
Impact of monopolar stimulation in three different models: a homogenous model with no anatomical detail, an anatomical model with electrode 3093 and an anatomical model with electrode 3889. The dark red shaded region indicates the −0.2 V isopotential volume and the red lines are the electric field lines

In the homogeneous model, there are no tissue boundaries surrounding the implanted electrode. Fig. 5 shows the homogeneous model with simulation results for the monopolar −1 V stimulation setting. The shaded area around the active contact is an isopotential surface set at −0.2 V and the lines are electric field lines originating on the active contact. In comparison, the presence of tissues with different conductivities changes this result. Fig. 5 shows two clear effects of the inhomogeneity with the two electrode models. First, the shape of the field changes as can be seen by the field lines, which are no longer symmetric around the electrode contact but are shaped by the tissue. Second, the isopotential volume changes somewhat with the introduction of the inhomogeneity in tissue conductance.

### 3.2 Monopolar settings

A critical consideration for both patients and clinicians is the issue of parameter selection and contact configuration. The first of the choices to be made in this process is whether to use a monopolar (single active contact on the electrode with the IPG as the return) or bipolar (two active contacts on the electrode) setting. We modelled both such settings to examine the difference in the induced VTA. Noting that the axons modelled run parallel to the electrode, we visualised the VTA as though looking down the long axis of the electrode, to show the region around the electrode where axons would be activated. The table also quantifies the differences in VTA by giving the percentage of axons activated in each case.

**Fig. 6:**
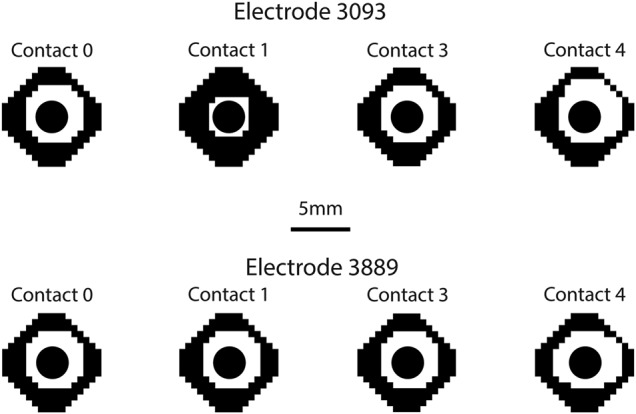
VTA plots for all monopolar configurations of both the 3093 and 3889 electrodes. The black region shows the points in space where the fibres did not fire in a 1:1 ratio with the SNS pulses. The white regions are the points where the fibres did fire in a 1:1 ratio

Fig. 6 shows the VTA plots for all monopolar settings, with changing cathode and for the two models of electrode. There are two notable differences between the sets of simulations. First, when the cathode is set to be the long contact 1 in the model 3093, the VTA is reduced by 15–20% (Table 1). This may seem counter intuitive, but given the orientation of the axons parallel to the electrode, this means the second spatial derivative of the potential distribution will be lower in this direction. The electric field as shown in Fig. 5 has field lines emanating from the active contact to the return contact. For monopolar settings, the return contact is the IPG. Hence, when a smaller contact is used, this electric field pattern will have more spatial difference compared to the large contact and consequently, the spatial derivative will be higher. Second, stimulation via contacts 3 in both models is less symmetric about the electrode and skewed. This is an effect of the inhomogeneity of the tissue in the vicinity of the contact.

**Table 1:** Percentage of the fibres modelled which are activated by each of the stimulation settings

Electrode model	Configuration	Activated contact	Percentage activation, %
3093	Monopolar	0	50
3093	Monopolar	1	35
3093	Monopolar	2	50
3093	Monopolar	3	60
3093	Bipolar	0	50
3093	Bipolar	1	40
3389	Monopolar	0	50
3389	Monopolar	1	50
3389	Monopolar	2	50
3389	Monopolar	3	55
3389	Bipolar	0	50
3389	Bipolar	1	50

### 3.3 Bipolar settings

**Fig. 7:**
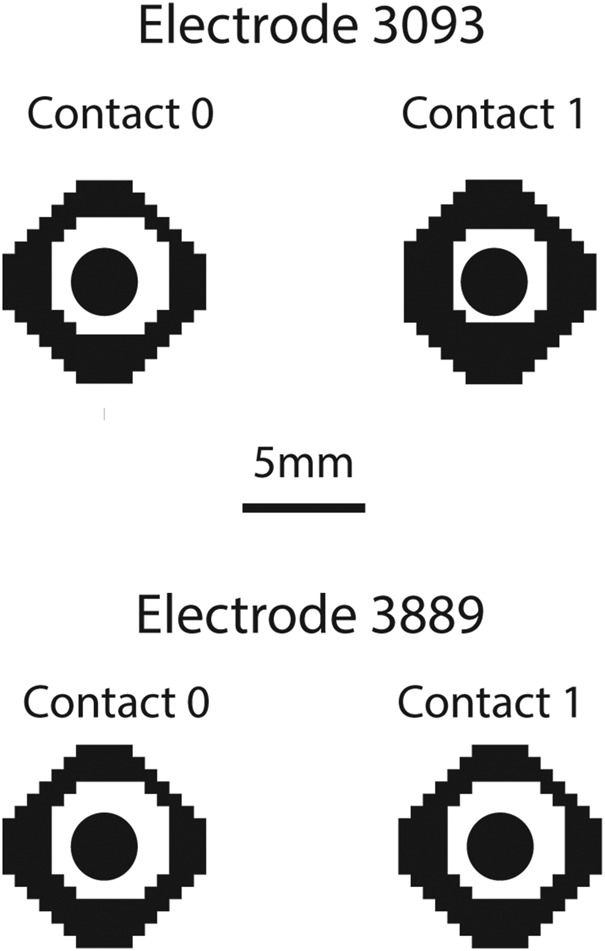
VTA plots for all bipolar configurations of both models of electrode, as described in Fig. 6

We simulated four bipolar settings, two for each electrode model and always via contacts 0 and 1, switching the cathode and anode. Once again the VTA plots in Fig. 7 demonstrate little difference in the VTA shape and size, with the exception being due to the long contact 1 on the 3093 electrode as shown in Fig. 6. When the long contact is used as a cathode, the VTA once again is smaller, this time by 10% compared to the other three biopolar settings. Once again, this is due to the shape of the electric field. Biopolar settings result in a dipole from the active contact to the return contact. When the larger contact is used this dipole is less focussed, and hence the second spatial derivative of the potential distribution will be reduced.

## 4 Discussion and conclusion

SNS is an effective clinical therapy used to treat faecal incontinence [19, 20]. However, the procedure has variable clinical benefit, induces side effects in a subset of patients and the process of optimising the parameters can be laborious, time consuming and difficult for the clinician and patient. These limitations are largely a result of the lack of information about the mechanisms of SNS and the lack of information about the spread of current in the tissue surrounding the implanted electrode.

This study has examined the SNS induced electric field in the pelvic region using a combined image segmentation method, finite element and biophysical modelling approach. Our model produced a number of interesting results. The first is that the anatomical detail of the tissues in the vicinity of the electrode is critical to understanding the induced field. While this result may be evident, in other neuromodulation paradigms such as DBS, tissue around the electrode only differs in electrical conductivity by ∼50%, unless the electrodes are located close to fluid filled regions [12]. In the case of SNS and this study, we have inhomogeneity of up to 500%. Indeed, here we considered only two anatomical features, the sacrum and the rectum. If more tissues were incorporated this may be a more pronounced effect [13].

The second main result was the comparison of the two models of electrode. The preference of which electrode to use has shifted recently from model 3093 back to 3889, which was one of the earlier models of electrode. We found that there was very little difference in the SNS induced field produced by the two models of electrode for all configurations apart from one sizable effect. Contact 1 on model 3093 has a length of 10.2 mm compared to the typical 3 mm contacts. Therefore, stimulation with this contact set as the cathode resulted in a 15–20% decrease in the effect of stimulation. Given the interest in designing new electrodes for neuromodulation [21–23] with new dimensions for individual contacts, appreciating the effects of such changes can be better understood with computational modelling.

The last aspect which we considered was contact configuration. This is a critical component of the procedure and one which impacts most heavily on the patient and clinician in terms of time and effort during follow up visits. Indeed, for SNS in particular, where the problem of initial lead positioning as well as lead migration can be significant [6], the need to select the ‘optimal’ parameters is paramount. Once again, given the direction of the axons relative to the electrode, we found little difference between monopolar and bipolar settings, even though in other neuromodulation modelling studies, the typical understanding is that monopolar stimulation spread much further bipolar stimulation [16]. Furthermore, the choice of active contact would be mainly based on the location of the electrode rather than any main difference between the effects of each contact as they all stimulated a similar amount of tissue.

In conclusion, this is the first computational model of SNS induced electric current flow to incorporate biophysical models of nerve fibres and to consider the specific impact of hardware and the clinically crucial aspect of contact configuration. The model could be refined with more detailed data of surrounding tissues and future work should also take into account time dependent stimulation parameters, such as frequency, which have been recently shown to play a role [24]. This may lead to better understanding of both therapeutic and adverse stimulation effects and enable patient-specific adjustments of stimulation parameters.

## 5 Funding and declaration of interests

Nada Yousif has received a research grant from Medtronic. Carolynne J. Vaizey and Yasuko Maeda have both received research grants and honorarium from Medtronic. Conflict of interest: None declared.
